# The Impact of Coexisting Asthma, Chronic Obstructive Pulmonary Disease and Tuberculosis on Survival in Patients with Lung Squamous Cell Carcinoma

**DOI:** 10.1371/journal.pone.0133367

**Published:** 2015-07-21

**Authors:** Jing-Yang Huang, Zhi-Hong Jian, Oswald Ndi Nfor, Kai-Ming Jhang, Wen-Yuan Ku, Pei-Chieh Ko, Shiou-Rung Jan, Chien-Chang Ho, Chia-Chi Lung, Hui-Hsien Pan, Yu-Chiu Liang, Yung-Po Liaw

**Affiliations:** 1 Department of Public Health and Institute of Public Health, Chung Shan Medical University, Taichung City, Taiwan; 2 Department of Neurology, Changhua Christian Hospital, Changhua, Taiwan; 3 Department of Physical Education, Fu Jen Catholic University, New Taipei City, Taiwan; 4 Department of Family and Community Medicine, Chung Shan Medical University Hospital, Taichung City, Taiwan; 5 Department of Pediatrics, Chung Shan Medical University Hospital, Taichung City, Taiwan; 6 School of Medicine, Chung Shan Medical University, Taichung City, Taiwan; 7 College of Humanities and Social Sciences, Taipei Medical University, Taipei City, Taiwan; University of Pittsburgh, UNITED STATES

## Abstract

**Background:**

Pulmonary diseases [asthma, chronic obstructive pulmonary disease (COPD), and tuberculosis (TB)] are associated with lung cancer mortality. However, the relationship between coexisting pulmonary diseases and survival in patients with lung squamous cell carcinoma (SqCC) has not been well defined.

**Methods:**

Patients newly diagnosed with SqCC between 2003 and 2008 were identified by linking the National Health Insurance Research Database and Taiwan Cancer Registry Database. Cases with SqCC were followed up until death, loss to follow-up, or study end in 2010. Information on health status, date of death and the main causes of death was ascertained from the National Death Registry Database. Cox proportional hazard regression was used to calculate the hazard ratio (HR) of coexisting asthma, COPD and/or TB.

**Results:**

During the study period, a total of 5406 cases with SqCC were enrolled. For all cause-mortality, HRs were 1.08 [95% confidence interval (CI), 0.99–1.18], 1.04 (95% CI, 0.97–1.12), and 1.14 (95% CI, 1.00–1.31) for individuals with asthma, COPD, and TB, respectively. Specifically, among men with coexisting pulmonary diseases, the HRs were 1.56 (95% CI, 1.23–1.97) and 1.11 (95% CI, 1.00–1.24) for individuals with asthma+COPD+TB and asthma+COPD, respectively. Among male patients with stage III SqCC, HRs were 3.41 (95%CI, 1.27–9.17) and 1.65 (95%CI, 1.10–2.47) for individuals with asthma+TB and asthma+COPD+TB, respectively. Among male patients with stage IV SqCC, HRs were 1.40 (95%CI, 1.00–1.97) and 1.25 (95%CI, 1.03–1.52) for individuals with asthma+ COPD+TB and asthma. Among female patients with stage I and II, HR was 0.19 (95%CI, 005–0.77) for individuals with asthma.

**Conclusions:**

Coexisting pulmonary diseases increased the risk of mortality from SqCC in male patients. For female patients with early stage SqCC, pre-existing asthma decreased mortality. These patients deserve greater attention while undergoing cancer treatment.

## Introduction

Squamous cell carcinoma (SqCC) accounts for approximately 20.3% of all lung cancers in Taiwan [1]. Therapeutic decisions in lung cancer have mostly been focused on characteristics such as stage of disease and performance status [2]. As the mean age in patients with non-small cell lung cancer (NSCLC) increases, the number of patients diagnosed with one or more other serious diseases increase at the time of diagnosis of lung cancer [3]. However, the burden of comorbidities and their impact on treatment has greatly been underrated.

Asthma, chronic obstructive pulmonary disease (COPD) and tuberculosis (TB) are the most common pulmonary comorbidities. The prevalence of asthma and COPD in Taiwan is 11.9% and 2.48%, respectively, while the incidence of TB is 54.5/100,000 [4–6]. The prevalence of asthma+COPD+TB, asthma+COPD, asthma+TB, COPD+TB, asthma, COPD and TB in patients with lung cancer is 0.8%, 7.2%, 0.2%, 1.3%, 5.1%, 14.7% and 0.9% [1]. However, only few studies with small sample sizes have evaluated the impact of specific comorbidities on stage-specific survival in NSCLC patients, and have yielded conflicting results. An analysis of 902 patients with early-stage (stage IA-IIB) NSCLC receiving surgical resection showed that COPD was associated with poor survival in patients with early-stage NSCLC [7]. Sekine et al. performed a retrospective review on 442 patients with stage IA NSCLC after complete resection and concluded that COPD was associated with poor long-term survival and high tumor recurrence [8]. Asthma also increased risk of lung cancer mortality [9]. The effect of TB on survival in patients with lung cancer remains controversial. Kuo and coworkers found that concomitant active TB prolongs survival in patients with stage III and IV NSCLC than those of NSCLC alone [10]. However, Leung et al. conducted a cohort of the elderly in Hong Kong and concluded that TB was independently associated with subsequent lung cancer mortality [11]. The aim of this study was to evaluate the association between coexisting pulmonary diseases (asthma, COPD, and/or TB) and mortality of SqCC patients.

## Material and Methods

### Data Source

All data in the National Health Insurance Research Database (NHIRD), Taiwan Cancer Registry Database (TCRD), and National Death Registry Database (NDRD) are anonymized and de-identified. Authors did not have any access to patients' identifying information. This study was approved by the Institutional Review Board of the Chung-Shan Medical University Hospital, Taiwan.

The National Health Insurance Program finances compulsory universal health care for 99% of 23 million residents in Taiwan [12]. The NHIRD contains all claims from diagnoses, prescriptions, and information on outpatient and inpatient care. Personal information including ethnicity, family history, lifestyle, occupation and habits such as smoking and alcohol use was not available in the NHIRD. The NHIRD is one of the largest datasets in the world and many epidemiological studies have been published in peer-reviewed journals [13, 14].

The study made use of data from individuals aged 20 years and above who were free from lung cancer before 2002. Subjects with incomplete data were excluded. Lung cancer cases were identified from 2003–2008 using the International Classification of Diseases, Ninth Revision, Clinical Modification (ICD-9-CM) code 162. The date of a subject’s diagnosis of lung cancer was defined as the index date. The cohort began in 2003 and subjects were followed up from the index date until death, loss to follow-up, or study end in 2010.

The SqCC was further confirmed by TCRD which was established in 1979. All major cancer care hospitals in Taiwan are obligated to submit cancer type, initial tumor stages and histology to the Taiwan Cancer Registry data sets, and all of the registries have been systemically converted to International Classification of Diseases, 9th or 10th Revision codes. Lung cancer was coded by ICD-9-CM 162 or ICD 10 C34.0, C34.1, C34.2, C34.3, C34.8, and C34.9 in TCRD. The morphological diagnosis of SqCC was made using the Ninth Revision of the International Classification of Diseases for Oncology, based on the codes; 80522, 80523, 80702, 80703, 80713, 80723, 80733, 80743, 80763, 80823, 80833 and 80843 for SqCC.

The NHIRD, NDRD and TCRD were used to retrieve information on the age of cancer onset, person-months of follow-up, death and survival time, and potentially unconfirmed cases diagnosed with cancer.

### Variables of Exposure

Demographic and comorbidity profiles were retrieved from the NHIRD. To confirm temporal relationship between comorbidities and all-cause mortality of SqCC, cases with asthma, COPD, TB and other comorbidities diagnosed 2 years before the index date were included in the study. The diagnoses of pulmonary diseases and other comorbidities were ascertained by either 2 outpatient visits or one hospitalization in one year. Baseline pulmonary diseases and other comorbidities identified in this study were asthma (ICD-9-CM: 493), COPD (ICD-9-CM: 490, 491, 492, 494, 496), TB (ICD-9-CM: 010–012, 137.0), chronic renal disease (ICD-9-CM: 585, 586), type II diabetes mellitus (ICD-9-CM: 250, excluding type 1 DM), hyperlipidemia (ICD-9-CM: 272), and smoking related cancers (ICD-9-CM: 140–150, 157, 160–161, 189).

Medications such as statins [15], inhaled or oral corticosteroids [16], and aspirin [17] have been associated with lung cancer. We identified patients who were prescribed inhaled and oral corticosteroids, statins, and aspirin from 2 years prior to index date to the end of the study.

### Statistical Analysis

All data analyses were performed using SAS 9.3 software (SAS Institute, Cary, NC). The number of person-months of follow-up was estimated from the index date until loss to follow-up, death or the termination of the study. All-cause mortality rates were defined as deaths per 100 person-months. The mortality rate, rate ratios and 95% confidence intervals (CIs) were estimated under the Poisson regression models. Multivariate Cox proportional hazards regression was performed to determine the strength of coexisting pulmonary diseases and SqCC mortality. In order to evaluate the effect of coexisting pulmonary diseases on all-cause mortality in patients with different stages of SqCC, three separate models were estimated for both genders: a model containing three pulmonary diseases (Model 1), a model containing pulmonary disease combinations (Model 2), and a count of pulmonary disease model (Model 3). All comparisons with a *P*-value < 0.05 were considered to indicate statistical significance.

## Results

Between 2003 and 2008, a total of 5406 patients were diagnosed with SqCC. The all-causes mortality among people diagnosed with SqCC included; SqCC, 79.5% (3715 cases), pneumonia, 0.3% (14), respiratory failure, 0.3% (12), sepsis, 0.2% (8), COPD, 0.3% (14), other cancers, 2.8% (133), and others 16.6% (775). Demographic and comorbidity profiles are listed in Table 1. Asthma, COPD, and TB increased risk for mortality in patients with SqCC with rate ratios of 1.12 (95% CI, 1.11–1.12), 1.08 (95% CI, 1.07–1.08), and 1.21 (95% CI, 1.20–1.22), respectively. The ever use of inhaled and oral corticosteroids, and statins decreased risk of all-cause mortality with rate ratios; 0.76 (95% CI, 0.75–0.76), 0.80 (95%CI, 0.79–0.80), and 0.62 (95%CI, 0.61–0.62), respectively. Aspirin use led to a slight increase of the rate ratio to 1.002 (95%CI, 1.001–1.003). Compared with women, there was increased risk of mortality in men with SqCC (rate ratio, 1.42; 95% CI, 1.41–1.42).

**Table 1:** Characteristics of patients with lung squamous cell carcinoma and all-cause mortality, Taiwan, 2003–2010.

	Duration (person-months)	No. of Death	Mortality rate (per 100 person-months) (95% C.I.)	Rate ratio (95% C.I.)
Asthma				
No (N = 4,508)	74,095	3,873	5.23 (5.22–5.23)	1
Yes (N = 898)	13,647	798	5.85 (5.83–5.86)	1.12 (1.11–1.12)
COPD				
No (N = 3,794)	62,297	3,241	5.20 (5.2–5.21)	1
Yes (N = 1,612)	25,445	1,430	5.62 (5.61–5.63)	1.08 (1.07–1.08)
TB				
No (N = 5,141)	8,3943	4,428	5.27 (5.27–5.28)	1
Yes (N = 265)	3,799	243	6.40 (6.34–6.45)	1.21 (1.20–1.22)
Sex				
Women (N = 629)	12,800	502	3.92 (3.91–3.94)	1
Men (N = 4,777)	74,942	4,169	5.56 (5.56–5.57)	1.42 (1.41–1.42)
Age of SqCC diagnosed				
<40 (N = 50)	930	43	4.62 (4.42–4.84)	1
40–59 (N = 1,022)	21,601	790	3.66 (3.65–3.67)	0.79 (0.76–0.83)
≧60 (N = 4,334)	65,211	3,838	5.89 (5.88–5.89)	1.27 (1.22–1.33)
Low income				
No (N = 5,269)	85,566	4,548	5.32 (5.31–5.32)	1
Yes (N = 137)	2,176	123	5.65 (5.56–5.74)	1.06 (1.05–1.08)
Stage				
I (N = 608)	20,954	328	1.57 (1.56–1.58)	1
II (N = 277)	7,273	194	2.67 (2.64–2.69)	1.70 (1.68–1.72)
III (N = 2,145)	35,736	1,874	5.24 (5.24–5.25)	3.35 (3.33–3.37)
IV (N = 2,376)	23,779	2,275	9.57 (9.56–9.58)	6.11 (6.08–6.15)
Surgery				
No (N = 4,496)	55,083	4,217	7.66 (7.65–7.66)	1
Yes (N = 910)	32,659	454	1.39 (1.38–1.40)	0.18 (0.18–0.19)
Comorbidities				
Diabetes				
No (N = 4,415)	71,501	3,802	5.32 (5.31–5.32)	1
Yes (N = 991)	16,241	869	5.35 (5.34–5.36)	1.01 (1.00–1.01)
Hyperlipidemia				
No (N = 4,497)	71,266	3,917	5.50 (5.49–5.50)	1
Yes (N = 909)	16,476	754	4.58 (4.56–4.59)	0.83 (0.83–0.84)
Chronic renal disease				
No (N = 5,232)	85,559	4,508	5.27 (5.26–5.27)	1
Yes (N = 174)	2,183	163	7.45 (7.38–7.56)	1.42 (1.40–1.43)
Smoking related lungcancers				
No (N = 5,153)	83,869	4,449	5.31 (5.30–5.31)	1
Yes (N = 253)	3,873	222	5.73 (5.68–5.78)	1.08 (1.07–1.09)
Geographical area				
Taipei (N = 1,450)	25,855	1,216	4.70 (4.69–4.71)	1
North (N = 657)	10,838	564	5.20 (5.19–5.22)	1.11 (1.10–1.11)
Central (N = 1,274)	20,033	1,123	5.61 (5.60–5.62)	1.19 (1.19–1.20)
South (N = 1,145)	17,974	986	5.49 (5.48–5.50)	1.17 (1.16–1.17)
Kaohsiung-Pingtung (N = 714)	1,1048	625	5.66 (5.64–5.68)	1.20 (1.20–1.21)
East (N = 166)	1,994	157	7.87 (7.78–7.97)	1.67 (1.65–1.70)
Urbanization				
Urban (N = 2,094)	36,599	1,776	4.85 (4.85–4.86)	1
Suburban (N = 2,110)	34,202	1,819	5.32 (5.31–5.32)	1.10 (1.09–1.10)
Rural (N = 1,202)	16,941	1,076	6.35 (6.34–6.36)	1.31 (1.30–1.31)
Ever use of medications between 2 years prior to index date and death				
Inhaled corticosteroids				
No (N = 4,155)	63,245	3,613	5.71 (5.71–5.72)	1
Yes (N = 1,251)	24,497	1,058	4.32 (4.31–4.33)	0.76 (0.75–0.76)
Oral corticosteroid				
No (N = 1,104)	14,650	937	6.40 (6.38–6.41)	1
Yes (N = 4,302)	73,092	3,734	5.11 (5.10–5.11)	0.80 (0.79–0.80)
Aspirin				
No (N = 3,298)	53,236	2,832	5.32 (5.31–5.32)	1
Yes(N = 2,108)	34,506	1,839	5.33 (5.32–5.34)	1.002 (1.001–1.003)
Statins				
No (N = 4,797)	7,4216	4,199	5.66 (5.65–5.66)	1
Yes (N = 609)	13,526	472	3.49 (3.48–3.50)	0.62 (0.61–0.62)

Abbreviation: CI, confidence interval; COPD, chronic obstructive pulmonary disease; TB, pulmonary tuberculosis.

Table 2 shows the adjusted hazard ratios (HRs) for all-cause mortality in SqCC patients. The HRs of pulmonary diseases were higher among SqCC patients with TB (HR, 1.14; 95%CI, 1.00–1.31). The HRs of ever use of medications were 0.91 (95%CI, 0.85–0.97) for inhaled corticosteroids, 0.70 (95%CI, 0.65–0.75) for oral corticosteroids, and 0.81 (95%CI, 0.73–0.89) for statins.

**Table 2:** Cox proportional model estimating the risk on all-cause mortality in patients with lung squamous cell carcinoma between 2003 and 2010.

	HR	95% C.I.	p-value
Asthma			
No	1		
Yes	1.08	0.99–1.18	0.053
COPD			
No	1		
Yes	1.04	0.97–1.11	0.283
TB			
No	1		
Yes	**1.14**	1.00–1.31	0.049
Sex			
Women	1		
Men	1.36	1.24–1.49	<0.0001
Age of SqCC diagnosis			
<40	1		
40–59	1.02	0.77–1.35	0.896
≧60	1.36	1.03–1.80	0.031
Low income			
No	1		
Yes	0.94	0.79–1.11	0.457
Stage			
I	1		
II	1.39	1.18–1.65	<0.001
III	1.94	1.72–2.19	<0.0001
IV	2.91	2.58–3.29	<0.0001
Surgery			
No	1		
Yes	0.36	0.32–0.40	<0.0001
Comorbidities			
Diabetes			
No	1		
Yes	1.09	1.01–1.17	0.032
Hyperlipidemia			
No	1		
Yes	0.93	0.86–1.01	0.070
Chronic renal disease			
No	1		
Yes	1.37	1.19–1.59	<0.0001
Smoking related cancers			
No	1		
Yes	1.11	0.99–1.23	0.080
Geographical area			
Taipei	1		
North	0.98	0.89–1.08	0.735
Central	1.02	0.94–1.11	0.641
South	1.05	0.96–1.15	0.283
Kaohsiung-Pingtung	1.06	0.97–1.17	0.196
East	1.15	0.98–1.36	0.093
Urbanization			
Urban	1		
Sub-urban	1.03	0.97–1.10	0.331
Rural	1.09	0.99–1.18	0.053
Ever use of medications between 2 years prior to index date and death			
Inhaled corticosteroid	**0.91**	0.85–0.97	0.005
Oral corticosteroid	**0.70**	0.65–0.75	<0.0001
Aspirin	1.04	0.98–1.11	0.158
Statins	**0.81**	0.73–0.89	<0.0001

Abbreviation: CI, confidence interval; COPD, chronic obstructive pulmonary disease; HR, hazard ratio; TB, pulmonary tuberculosis.

**Fig 1:**
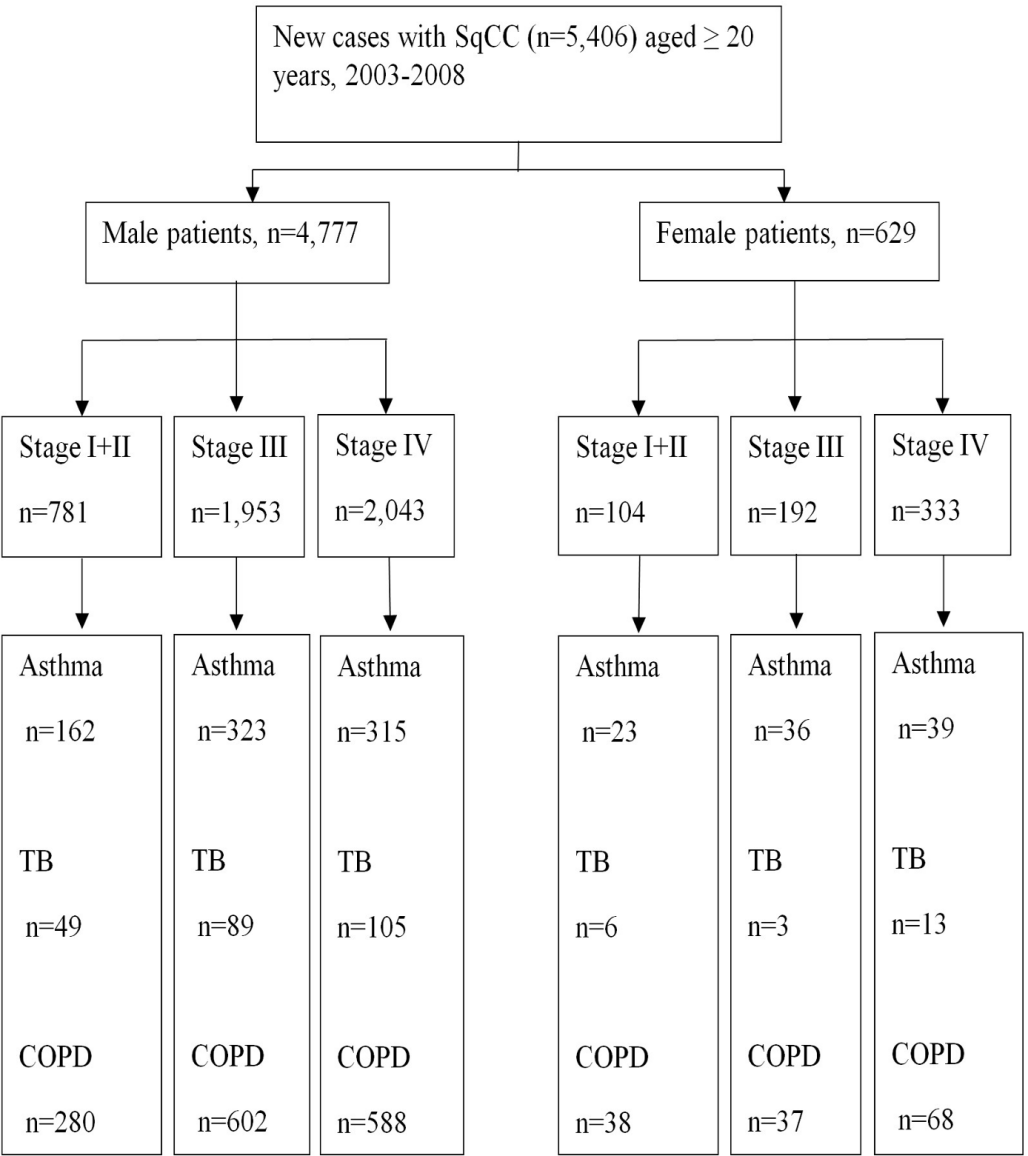
Study design flow chart. Abbreviation: COPD, chronic obstructive pulmonary disease; SqCC, squamous cell carcinoma; TB, tuberculosis.

Fig 1 shows number of patients with SqCC stratified by sex, lung diseases and stage. Table 3 illustrates the coexisting pulmonary diseases, cancer stages, and the HRs of all-cause mortality of SqCC by gender after adjusting for confounding factors. For different combinations of pulmonary diseases among all SqCC patients, the HRs were higher among male individuals with asthma+COPD+TB (HR, 1.56; 95% CI, 1.23–1.97) and asthma+COPD (HR, 1.11; 95% CI, 1.00–1.1.24) as shown in Model 2. The HRs increased in male patients with two (HR, 1.11; 95% CI, 1.01–1.22) or three (HR, 1.56; 95% CI, 1.23–1.97) pulmonary diseases as shown in model 3. Specifically, among male patients with stage III, HRs were 1.32 (95% CI, 1.05–1.65) for TB (Model 1), and 3.41 (95% CI, 1.27–9.17) for asthma+TB and 1.65 (95%CI, 1.10–2.47) for asthma+COPD+TB (Model 2). Among male patients with stage IV, HRs were 1.40 (95%CI, 1.00–1.97) for asthma+ COPD+TB and 1.25(95%CI, 1.03–1.52) for asthma (Model 2), and 1.14 (95%CI, 1.02–1.26) for any lung diseases (Model 3). Among female patients with stage I and II, asthma decreased mortality of SqCC at HR of 0.19 (95%CI, 0.05–0.77). However, there was no significant association between mortality and pulmonary diseases in female patients with stage III and IV SqCC when stratified by models.

**Table 3:** Estimated hazard ratios of all-cause mortality related to pulmonary diseases and stage of lung squamous cell carcinoma.

	**HR (95% C.I.)**							
	**All stages**		**Stage I+II**		**Stage III**		**Stage IV**	
	Male	Female	Male	Female	Male	Female	Male	Female
**Model 1**								
Asthma	1.14(0.99–1.31)	1.14 (0.87–1.48)	1.10 (0.88–1.37)	0.81 (0.36–1.84)	1.09 (0.95–1.25)	1.19 (0.74–1.90)	1.09 (0.95–1.24)	1.24 (0.85–1.82)
COPD	1.14 (0.99–1.31)	0.93 (0.74–1.17)	1.07 (0.88–1.31)	1.20 (0.54–2.67)	1.03 (0.93–1.15)	0.92 (0.57–1.48)	1.06 (0.96–1.18)	0.79 (0.58–1.09)
TB	1.06 (0.98–1.13)	1.06 (0.63–1.79)	0.95 (0.67–1.37)	0.45 (0.09–2.32)	**1.32 (1.05–1.65)**	2.81 (0.76–10.3)	1.07 (0.88–1.31)	1.16 (0.60–2.24)
**Model 2**								
None	1	1	1		1	1	1	1
Asthma	1.09 (0.95–1.24)	0.96 (0.66–1.38)	0.84 (0.59–1.2)	**0.19 (0.05–0.77)**	1.02 (0.83–1.27)	0.98 (0.53–1.82)	**1.25 (1.03–1.52)**	1.29 (0.76–2.17)
COPD	1.06 (0.98–1.15)	0.86 (0.65–1.14)	0.93 (0.73–1.19)	0.61 (0.24–1.53)	1.04 (0.91–1.17)	0.79 (0.45–1.41)	1.10 (0.97–1.23)	0.86 (0.59–1.25)
TB	1.11 (0.83–1.49)	3.02 (0.72–12.66)	0.51 (0.23–1.16)	-	1.37 (0.87–2.15)	-	1.28 (0.84–1.96)	3.21 (0.75–13.84)
Asthma+ COPD	**1.11 (1.00–1.24)**	1.23 (0.86–1.75)	1.19 (0.91–1.57)	1.77 (0.59–5.25)	1.13 (0.96–1.33)	1.33 (0.68–2.63)	1.10 (0.93–1.29)	0.94 (0.55–1.60)
Asthma + TB	0.85 (0.48–1.5)	1.50 (0.36–6.19)	-	-	**3.41 (1.27–9.17)**	3.58 (0.37–34.5)	0.64 (0.32–1.29)	1.30 (0.18–9.52)
COPD+ TB	1.13 (0.91–1.39)	0.84 (0.41–1.73)	0.97 (0.57–1.65)	0.80 (0.15–4.19)	1.18 (0.85–1.64)	-	1.13 (0.82–1.55)	0.67 (0.29–1.57)
Asthma+COPD+TB	**1.56 (1.23–1.97)**	0.96 (0.37–2.51)	1.71 (0.96–3.04)	0.02 (0.00–3.61)	**1.65 (1.10–2.47)**	3.05 (0.63–14.86)	**1.40 (1.00–1.97)**	2.14 (0.50–9.23)
**Model 3**								
None	1	1	1	1	1	1	1	1
Any one of lung diseases	1.07 (0.99–1.15)	0.91 (0.72–1.15)	0.87 (0.71–1.08)	**0.40 (0.17–0.93)**	1.05 (0.93–1.17)	0.88 (0.57–1.37)	**1.14 (1.02–1.26)**	0.99 (0.72–1.36)
Any two of lung diseases	**1.11 (1.01–1.22)**	1.15 (0.83–1.57)	1.15 (0.89–1.48)	1.23 (0.50–3.02)	1.15 (0.99–1.34)	1.43 (0.75–2.75)	1.07 (0.93–1.24)	0.85 (0.54–1.34)
Asthma+COPD+TB	**1.56 (1.23–1.97)**	0.95 (0.37–2.49)	1.70 (0.95–3.02)	0.03 (0.00–2.27)	**1.65 (1.10–2.47)**	3.24 (0.67–15.66)	**1.40 (1.00–1.96)**	2.07 (0.48–8.90)

Each model was adjusted by age of SqCC diagnosis, low income, surgery, comorbidities, geographical area, urbanization, and medications.

Abbreviation: CI, confidence interval; COPD, chronic obstructive pulmonary disease; HR, hazard ratio; TB, pulmonary tuberculosis.

## Discussion

The impact of coexisting pulmonary diseases on the mortality in patients with different stages of SqCC have not been established. To the best of our knowledge, this is the first study to investigate the relationship using large sample size. This study demonstrates that coexisting asthma, COPD and TB increased mortality risk in male patients with SqCC. For female patients with early stage SqCC, pre-existing asthma decreased mortality.

There are conflicting data on the association between asthma and lung cancer. The mechanism linking them is unknown. Doris et al. conducted ovalbumin induced allergic airway inflammation and chemical carcinogenesis in a murine model [18]. They found that ovalbumin-induced allergic inflammation during tumor initiation, progression, or continuously did not impact the number, size, or histologic distribution of urethane-induced pulmonary neoplastic lesions. It suggests that not all types of airway inflammation influence lung carcinogenesis. El-Zein et al. analyzed 3,300 Canadian cancer cases and 512 controls who reported a prior medical diagnosis of asthma, medication use, and several covariates, and found that a history of asthma was negatively associated with all cancer types combined [19]. In a follow up study by El-Zein et al., a diagnosis of asthma had a decreased odds ratio (OR, 0.90; 95% CI, 0.65–1.24) of developing lung cancer, which decreased to 0.76 (95% CI 0.54–1.08) for subjects whose onset was more than 2 years before lung cancer diagnosis or interview and then to 0.64 (95% CI 0.44–0.93) when restricted to subjects who reported using medication for their asthma [20]. The authors suggested mechanistically that in those with asthma related hyperreactive immune system might lead to a more efficient elimination of abnormal cells, thus lowering lung cancer risks. They also suggest that there may be genetic differences in lung cancer susceptibility regionally. It is consistent with our findings that asthma decreases mortality of female patients with stage I+II SqCC.

The survival of asthmatic and non-asthmatic lung cancer patients appeared similar after matching for sex, stage, histological type, and age in Finland during 1970–1989 [21]. Brown et al. analyzed a nationally representative sample of 9087 adults aged 30–75 years to estimate asthma related death from lung cancer and showed that asthma increased risk of lung cancer mortality among nonsmokers with a relative risk of 3.54 (95% CI: 1.93–6.42) [9]. No studies have reported the association between pulmonary diseases and the mortality in patients with SqCC. Our study showed that asthma was specifically associated with the mortality in male patients with SqCC in stage IV.

Lung cancer is closely related to COPD [22]. Higher COPD grades had higher rates of cancer-related deaths after lung cancer surgery [23]. Sekine et al. showed that the impact of COPD on survival was clear only in stage 1A and had a higher incidence of tumor recurrence [8]. However, the impact of COPD on the mortality of SqCC has not yet been investigated. Stages III and IV was clearly predominant in cases with SqCC in our database. Our finding showed that COPD specifically did not increase risk of mortality in SqCC patients.

The effect of TB on subsequent lung cancer mortality increased with adjusted HR of 2.01 (95%CI, 1.40–2.90) in elderly patients in Hong Kong [11]. Moreover, this study did not include the stage, histologic type, and surgery of cases with lung cancer. Zhou et al. analyzed 64 cases with old pulmonary lesion in 782 NSCLC patients receiving surgical resection and concluded that old TB lesion increased mortality risk in patients with SqCC (HR, 1.72; 95% CI, 1.12–2.64) [24]. However, Kuo et al in a study analyzing 276 cases with NSCLC (stage III and stage IV) reported that the survival of SqCC with concomitant active TB was better than adenocarcinoma or undetermined NSCLC with TB [10]. Their samples were therefore not representative of the general SqCC population. In contrast to previous studies, the larger number and more representative nature of the samples collected in our study provide a more reliable statistical power for assessing the relationship between SqCC and TB. In our study, the effect of TB on overall SqCC mortality is somewhat attenuated when stratifyied by gender due to relative small sample size. Besides, TB is an independent predictor of poor survival in male patients with SqCC in stage III.

Many elderly patients with obstructive airway disease have features of both asthma and COPD, termed asthma-COPD overlap syndrome, whose prevalence in Italy was 1.6%, 2.1% and 4.5% in the 20–44, 45–64 and 65–84 age groups, respectively [25]. The overlap syndrome has been associated with systemic inflammation, greater decline in lung function, more respiratory exacerbations, a decreased quality of life, and increased mortality and health care utilization than those with asthma or COPD alone [26–28]. However, this is not a well-defined syndrome and there is no specific ICD-9 codes for asthma-COPD overlap syndrome. Classifying the patient as an “asthma-COPD” overlap would involve more extensive clinical evaluation and testing. The co-morbidities of COPD and asthma were associated with lung cancer survival [29]. Moreover, Tammemagi also reported that TB was also associated with lung cancer survival [29]. Inghammar et al. reported that active TB increased risk of death in COPD patients from all causes compared to the control subjects with TB [odds ratio (OR), 2.2; 95% CI, 1.2–4.1] [30]. The coexistence of pulmonary diseases increase SqCC risk in men. The HRs were 3.98 (95% CI, 3.22–4.93), 2.68 (95% CI, 2.45–2.93), and 2.01 (95% CI, 1.14–3.54) for individuals with asthma+COPD+TB, asthma+COPD, and asthma+TB, respectively [1]. In our study, coexistence of asthma, COPD, and TB increased risk of mortality in male patients with SqCC in stages III and IV. While the exact mechanisms of these associations have not been delineated, it may be explained by biologically additive effects of systemic inflammatory processes of the lung and compromised immune clearance of Mycobacterium tuberculosis [31, 32]. This situation could predispose to carcinogenesis, decreased clearance of tumor cells and poor survival.

Our results showed that coexisting pulmonary disease may exert direct effects, hence increasing the risk of mortality of SqCC in men, but not women. There were not enough female patients to accurately analyze. More studies would need to be done before the association between pulmonary diseases and mortality in female SqCC patients can be concluded. Since information on smoking was not available whereas smoking is almost ten times more prevalent in men (45.7%) than women (4.8%) in Taiwan [33], this might have influenced the observed differences in survival between men and women. Continued smoking after diagnosis of lung cancer has been associated with a significantly increased risk of all causes of mortality (HR, 2.94; 95% CI, 1.15–7.54), tumor recurrence (HR, 1.86; 95% CI, 1.01–3.41) in early stage NSCLC, and development of a second primary tumor (HR, 4.31; 95% CI,1.09–16.98) [34].

Besides, sex hormones play a role in gender-based differences that may contribute to pathogenesis of disease or serve as protective factors [35]. Female gender exerted a positive effect on disease-related survival in patients with NSCLC receiving resection [36]. The expression of estrogen receptor –β is more frequent in lung tissue in female patients with NSCLC and correlate with grade of differentiation, lymph node metastasis and survival [37]. Epidermal growth factor receptor (EGFR) is a receptor tyrosine kinase involved in pathways leading to cell growth, differentation, and proliferation [38]. Estrogen receptor interacts with the downstream mediators of EGFR signaling in NSCLC [39], and that mutation of EGFR correlate with clinical responsiveness to the tyrosine kinase inhibitor [40]. These important differences favor women in terms of response to therapy and long-term survival after the diagnosis of lung cancer, regardless of histology or stage [41].

Asthma and COPD are chronic inflammatory diseases and are associated with lung carcinogenesis [42]. The use of inhaled corticosteroids is associated with a reduced risk of lung cancer (OR, 0.79; 95%CI, 0.69–0.90) [16]. Inhaled corticosteroids also reduce the risk of lung cancer among former smokers with diagnosed COPD [43]. Statin may limit the availability of cholesterol required for tumor cell proliferation and metastasis [44], and simvastatin at a defined daily dose of over 150 reduced lung cancer risk compared with non-users (OR, 0.77; 95% CI, 0.62–0.97) in women [15]. The use of statin before a cancer diagnosis is associated with reduced cancer-related mortality [45]. Results from those studies are consistent with our findings. Although aspirin prevented distant metastasis and had early reduction in cancer deaths in trials of daily aspirin versus control [46], we did not find any association between aspirin and SqCC.

The strengths of this study were numerous. First, our study was a retrospective cohort study with a large sample size and long follow-up to minimize any concern over reverse causality. Small sample size limited reliability of previous study and gender-specific analysis of risk factors. Second, our study included all stages of lung SqCC without limiting to surgically resected lung cancer patients. Third, there was completeness of lung cancer cell type ascertainment from database. Fourth, with at least 2 years between diagnosis of pulmonary diseases and index date, hence the chances of misclassifying early lung cancer as asthma, COPD, or TB were few.

This paper presents some limitations. First, possible prognostic factors, such as performance status, visceral pleural invasion, and lymphovascular invasion were not available and these factors could have affected data analysis. Second, smoking is a common risk for lung cancer and COPD. When looking at COPD and lung cancer risk, pack years of smoking is critical. Smoking is not available in the NHIRD, TCRD and NDRD. Third, information regarding ethnicity and occupation was not available in the database. Fourth, the lack of significance between pulmonary diseases and mortality in female patients with SqCC may be attributable to the relatively small sample size. More studies would need to be done before solid conclusions can be drawn for females. Fifth, laboratory findings, including methacholine challenge test results or bronchodilator reversibility on spirometry were not available in the NHIRD. Some of the patients labeled as “asthma + COPD” may have had only COPD or asthma. The accuracy of diagnosis may vary depending on the health care professionals. Because of the privacy rule protecting care providers, it was impossible to verify who (general practitioners or pulmonary specialists) made these diagnoses from the NHIRD. Sixth, adjustments were not made for the dose of inhaled and oral corticosteroid. Further studies are needed to evaluate the dose effect of medications on SqCC mortality.

In conclusion, this study shows that this association can be of deleterious prognostic value in male patients with SqCC and coexisting pulmonary diseases. Among female patients with early stage SqCC, pre-existing asthma was associated with decreased mortality. Because of aging and high prevalence of asthma, COPD, and TB, efforts to reduce mortality of SqCC should be directed towards optimizing the management of coexisting pulmonary diseases.
